# Mycotoxins’ Activity at Toxic and Sub-Toxic Concentrations: Differential Cytotoxic and Genotoxic Effects of Single and Combined Administration of Sterigmatocystin, Ochratoxin A and Citrinin on the Hepatocellular Cancer Cell Line Hep3B

**DOI:** 10.3390/ijerph110201855

**Published:** 2014-02-07

**Authors:** Nikolia Αnninou, Ekaterini Chatzaki, Fotini Papachristou, Μichail Pitiakoudis, Constantinos Simopoulos

**Affiliations:** 1 Cell Culture Unit, Laboratory of Experimental Surgery and Surgical Research, Medical School, Democritus University of Thrace, Alexandroupolis 68100, Greece; E-Mails: fpapachr@med.duth.gr (F.P.); simop@med.duth.gr (C.S.); 2 Laboratory of Pharmacology, Medical School, Democritus University of Thrace, Alexandroupolis 68100, Greece; E-Mail: achatzak@med.duth.gr; 3 2nd Surgical Department, University General Hospital of Alexandroupolis, Medical School, Democritus University of Thrace, Alexandroupolis 68100, Greece; E-Mail: mpitiak@med.duth.gr

**Keywords:** sterigmatocystin, ochratoxin A, citrinin, metabolic activity, genotoxicity, cytostaticity, cytotoxicity, picoMolar concentrations

## Abstract

Food safety organizations indicate the likelihood of constant human and animal exposure to mycotoxin mixtures as a possible negative public health impact. Risk assessment demonstrates that certain mycotoxins of *Aspergillus* and *Penicillium* spp. are toxic and hold a significant genotoxic efficacy at nanomolar concentrations. The aim of the current study was to investigate the potential cytogenetic effects of sterigmatocystin (STER), ochratoxin A (OTA) and citrinin (CTN) alone or in combination, at pM to μΜ concentrations, on the human hepatocellular cancer cell line Hep3B. MTT reduction, mitotic divisions, cell cycle delays and sister chromatid exchange rates (SCE) were determined as endpoints of metabolic activity, cytotoxicity, cytostaticity, and genotoxicity, respectively. All mycotoxin treatments induce SCE rates from 10^−12^ M, while their cytotoxic and cytostatic potential varies. In PRI and MI assays, but not at MTT, STER alone or in combination with OTA + CTN appeared cytostatic and cytotoxic, even at 10^−12^ M, while CTN alone and all other combinations displayed substantial cellular survival inhibition in doses ≥ 10^−8^ M. Co-administration of STER + OTA or STER + CTN in concentrations ≤ 10^−1^ M, increased the MI and MTT activity, while it did not affect the PRI. Mycotoxin co-treatments revealed in general similar-to-additive or antagonistic genotoxic and cytotoxic effects. Our results for the first time describe that STER alone or in combination with OTA and/or CTN share a cytotoxic and cytogenetic potential even at picoMolar concentrations on human hepatoma cells *in vitro.*

## 1. Introduction

Mycotoxins are secondary metabolic products of molds, whereby the major mycotoxin-producing fungi are certain *Aspergillus, Fusarium and Penicillium* species [1]. They are produced pre- and post-harvesting under favorable environmental conditions (temperature, humidity) in a wide range of agricultural commodities [2,3]. According to surveys, mycotoxins (in native and metabolized conjugated form) possess potentially toxic and even carcinogenic (after long-term exposure) properties [1,2,3,4,5,6,7]. Their impact on public health depends on the consumption of mycotoxin-contaminated processed agricultural and animal-derived products or meat [2,4]. 

Sterigmatocystin (STER) is a potentially health hazardous mycotoxin, mainly produced by various *Aspergillus, Bipolaris* and *Penicillium luteum* species. As a natural contaminant, it is often detected in food and feed [8,9,10], while recent data indicate that exposure to STER might also occur through inhalation or direct skin contact [10]. STER, a precursor of aflatoxin biosynthesis with hepatotoxic action, is classified in Group 2B (possibly carcinogenic) by the International Agency of Research in Cancer [11,12,13]. According to *in vivo* as well *in vitro* studies, it was more cytotoxic than aflatoxin in human adenocarcinoma lung cells A549 and human esophageal epithelial cells Het-1A and exhibited a mutagenic potential [14].

Ochratoxin A (OTA), produced by several fungi strains of *Aspergillus* and *Penicillium,* belongs to the major classes of mycotoxins [15]. It is characterized as possibly carcinogenic to humans (Group 2B according to the IARC classification) and is found in many food commodities and animal feeds as a mixture with other mycotoxins, in its native or “disguised” and undetectable forms [7,15]. In addition to being nephrotoxic and renal carcinogenic, adequate studies on animals revealed that it could also severely affect liver function and even lead to hepatocellular cancer [15,16,17,18]. 

Citrinin (CTN) is mainly produced by several fungal strains belonging to the genera *Penicillium*, *Aspergillus*, and *Monascus*, including *Penicillium verrucosum* strains that also produce OTA. IARC evaluated the carcinogenic properties of CIT and classified it in Group 3 (not classifiable as to its carcinogenicity to humans) [19,20]. Many publications indicate the frequent co-occurrence of OTA and CTN in the environment, as well as the possible involvement (after long-term exposure) of these two mycotoxins to the development of Balkan Endemic Nephropathy (BEN) [21,22,23]. CTN has demonstrated dose-dependent cytotoxic properties *in vitro* and a controversial genotoxic profile [19,20].

There is a high probability for humans and animals to be perpetually exposed to mycotoxins, through an additive process, due to tissue and food chain accumulation of these substances or their metabolites. Food processing and baking does not eliminate mycotoxins completely [17]. Studies have detected 440 pM–1 nM of CIT, 0.9–1.3 nM of OTA and about 30 pM of STER, in human serum of healthy individuals through normal exposure [24,25,26,27,28]. It has been reported that fungi strains of *Aspergillus* and *Penicillium*, from which OTA, CTN and STER are mainly produced, usually coexist, increasing the likelihood that these toxins can be present at the same time in agricultural commodities [29]. 

The aforementioned results motivated us to investigate the *in vitro* effects of pM to μM concentrations of these three mycotoxins, individually or in combination, in the human hepatocellular cancer cell line Hep3B. Sister chromatid exchanges (SCE), mitotic divisions (mitotic index, MI), cell cycle delays (proliferation rate index, PRI) and MTT reduction served as endpoints of genotoxicity, cytotoxicity, cytostaticity and metabolic activity (cell viability), respectively. The Hep3B cell line was chosen because, as a member of hepatoma cell line group, it retains metabolic properties normally lost during culture of primary or lymphocyte cells and is therefore considered acceptable for toxicity studies [30,31,32]. 

## 2. Materials and Methods

### 2.1. Chemicals

Dulbecco’s MEM (high glucose), trypsin-EDTA solution, fetal bovine serum (FBS), colcemide and penicillin/streptomycin solution (10000:10000) were obtained from GIBCO (Carlsbad, CA, USA). 5-bromo-2’-deoxyuridine (BrdU) and bisbenzimide H33258 were obtained from Applichem (Darmstadt, Germany). Dimethyl sulfoxide (DMSO), 3-(4,5-dimethylthiazol-2-yl)-2,5-diphenyltetrazolium bromide (MTT), ochratoxin A, citrinin and sterigmatocystin were obtained from Sigma-Aldrich Co. (St. Louis, MO, USA). Ochratoxin A, citrinin and sterigmatocystin stock solutions were prepared in DMSO and then further diluted with complete culture medium to the desired concentrations. 

### 2.2. Cell Culture

Human hepatocellular carcinoma cell line Hep3B was kindly provided by Professor G. Kolios (Department of Pharmacology, Faculty of Medicine, Democritus University of Thrace, Greece). The cells were maintained in Dulbecco’s MEM supplemented with 10% FBS and 1% penicillin/streptomycin solution, in a 37 °C humidified incubator under an atmosphere of 5% CO_2_. Passages 20–30 were used for all experiments. On attaining 75%–80% confluency, the cells were trypsinized and were seeded in appropriate cell numbers depending on the type of the experiment. All experiments took place 24 h after seeding. 

### 2.3. MTT Assay

Metabolic activity was determined by MTT assay. Cells were seeded at a density of 10^4^ cells/well in 96-well plates. Cells were treated with various concentrations of STER, OTA and CTN for 24 h and 48 h. Control cultures were treated with drug-free vehicle DMSO (0.1%). Each treatment was assessed in 8 replicate cultures, in three independent experiments. At the end of the incubation period, the medium was discarded and each well received 200 μL of fresh medium for one hour, at 37 °C. After that, 20 μL of MTT (5 mg/mL in PBS) were added to all wells, for four hours at 37 °C. Formazan crystals were dissolved by adding 100 μL 0.04 M HCL/isopropanol, for 15 min at 37 °C and the optical density was determined at 570 nm using a microplate reader (ExpertPlus, ASYS Hitech GmbH, Nordstrasse 4. A-5301 Eugendorf, Austria). Data are presented as mean of MTT reduction ± standard error (SE) between the replicates (n = 3). IC_50_ values were calculated using GraphPad Prism 5.91 (GraphPad Software, San Diego, CA, USA) and are expressed as mean IC_50_ ± standard deviation (SD).

### 2.4. SCE Assay

For SCE determination 2 × 10^5^ cells were used. Hep3B cells were treated with mycotoxins at concentrations ranging from 10^−12^–10^−6^ Μ and 4 μg/mL BrdU for 72 h. Untreated cells and cells treated with 0.1% DMSO served as controls. After 48h of culture 0.07 μg/ml of colcemide was added for 24h and at the end of incubation period cultures were harvested with hypotonic KCl solution and fixed in methanol-acetic acid (3:1, v/v). All treatments were studied in three independent experiments. Differential staining of sister chromatids was achieved by a modified Fluorescence Plus Giemsa (FPG) technique [33]. 

Scoring was performed in a blind fashion. SCE rates—a quantitative and qualitative index of genotoxicity—were evaluated in a total of 60–75 second-division metaphases for each treatment. Since the number of chromosomes in Hep3B cells varies (modal number = 60) the frequency of SCE/chromosome was determined. The proliferation rate index (PRI)—a qualitative index of cytostaticity—was calculated according to the formula:

PRI = (M_1_ + 2 × M_2_ + 3 × M_3+_) / N

where M_1_, M_2_ and M_3+_ correspond to the number of metaphases in the first, second, and third or subsequent mitotic divisions, respectively, while N is the total number of metaphases scored [34]. A total of 500–600 cells were scored for each treatment. To determine the mitotic index (MI)—a qualitative index of cytotoxicity—a total of about 12,000 nuclei were scored for each treatment. MI was expressed as the number of metaphases per 1000 nuclei (‰). 

The expected effects (EV)—as if the mycotoxins were acting independently and additively—of the combined treatments on SCE rates were estimated by the following formula:

EV = (OV_1_ + OV_2_ + OV_3_) – (N − 1) × OV_control_

where, EV is the expected value of the combined treatment of N substances, OV is the mean observed value of each individual treatment of N substances and OV_control_ is the mean observed value of control cultures [35]. If the OV of a combined treatment is lower, similar to or greater than the EV, substances are considered to have an antagonistic, additive or synergistic effect, respectively.

### 2.5. Statistical Analysis

Data are presented as mean ± standard error of mean (SEM). SCE frequencies were logarithmically transformed prior further analysis. One way analysis of variance (ANOVA) followed by Bonferroni's *post hoc* test was employed for multiple comparisons and unpaired Student’s t-test for individual comparisons. Statistical analysis was performed by SPSS 16.0 (SPSS Inc., Chicago, IL, USA). All tests were two-tailed, while *p* values < 0.01 were considered statistically significant. The statistically significant (*p* < 0.05) differences between expected and observed values were estimated by an unpaired t-test. Additive effect is seen when there is no difference between expected and observed values. Synergism means that the observed values are significantly above the expected values and antagonism represents the exact opposite.

### 2.6. Coefficient of Drug Interaction (CDI) Assessment

The evaluation of the nature of mycotoxin interactions when applying the MTT assay was calculated using the coefficient of drug interaction (CDI) index calculated as CDI = AB/(A × B). According to the average optical density (*OD_570_*) of each group that we obtained by performing the MTT assay, AB is the ratio of the mycotoxin combination (CTN/OTA, CTN/STER, STER/OTA, STER/OTA/CTN) to the respective control group (AB = AB_OD570_/Control AB_OD570_) in *OD_570_*; Similarly, A or B is the ratio of the single agent to the respective control group (A= A _OD570_/Control A _OD570 _and B = B _OD570_/ Control B _OD570_) in *OD_570_*. Thus, CDI value <, =, or > than 1 indicates synergistic, additive or antagonistic interaction, respectively [23].

## 3. Results

### 3.1. Effect of CTN, OTA and STER on MTT Reduction in Hep3B Cells

**Figure 1:**
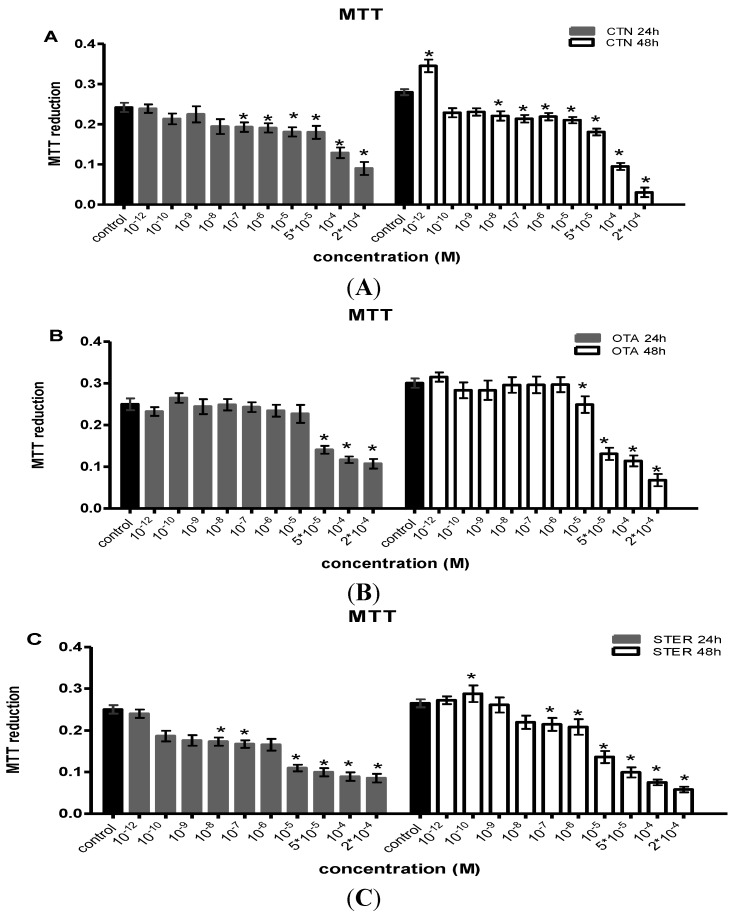
MTT reduction as a measure of metabolic activity in Hep3B cells after single administration of (**A**) citrinin (CTN) (**B**) ochratoxin Α (OTA) and (**C**) sterigmatocystin (STER), for 24 h and 48 h; Bars represent mean values of every concentration at each time point, which was evaluated at three individual experiments, performing measurements at a number of 8-plicates each time (8-plicates, n = 3).

Note: Error bars give the standard error (SE) between the replicates (n = 3). The asterisks represent statistically significant differences over the respective vehicle-treated cells (control) ( * *p* < 0.01).

The effects of CTN, OTA and STER administered in a single dosage on Hep3B cells as measured by the MTT assay are shown in Figure 1. 

STER and CTN exhibited cell toxicity over a period of two days, causing inhibition of MTT-reducing activity in concentrations equal or higher than 10^−8 ^M. For OTA the same effect is obvious in concentrations higher than 10^−6 ^M. IC50 values for these actions are presented in Table 1. STER has the lowest IC50 in comparison with OTA and CTN for all two time points examined. Interestingly, a biphasic dose-dependent action of STER and CTN was observed at 48h, as in lower concentrations, a statistically significant increase in MTT activity was shown.

**Table 1:** Mean values of IC50 in micromolar (μM) ± SD for CTN, OTA, STER as determined by MTT assay, were obtained using non-linear regression of three independent experiments in 8-plicates through the GraphPad Prism 5.91 package.

	24 h	48 h
	IC_50_ (μM) ± SD	IC_50_ (μM) ± SD
**CTN**	124 ± 4.4	77 ± 2.3
**OTA**	104 ± 3.1	45 ± 1.4
**STER**	58 ± 3.1	22 ± 0.7

### 3.2. Effect of CTN, OTA and STER Co-Administration on MTT Reduction in Hep3B Cells

**Figure 2:**
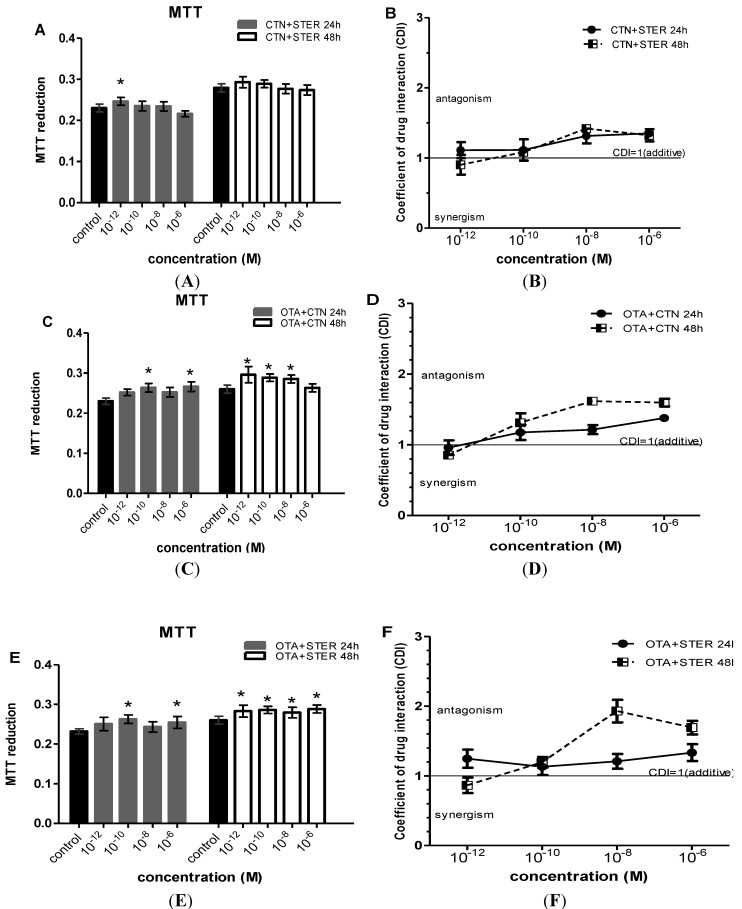
Evaluation of MTT activity in Hep3B cells after co-administration of (**A**) CTN and STER (**C**) OTA and CTN (**E**) STER and OTΑ for 24 and 48h; Bars represent mean values of every concentration at each time point, which was evaluated at three individual experiments, performing measurements at a number of 8-plicates each time (8-plicates, n = 3). Error bars give the standard error (SE) between the replicates (n = 3). The asterisks represent statistically significant differences over the respective vehicle-receiving cells (control) (* *p* < 0.01). CDI values (CDI = 1 CDI<1 and CDI>1, shows additive, synergic and antagonistic action respectively) after co-administration of (**B**) CTN and STER (**D**) CTN and OTA (**F**) OTA and STER. Again error bars give the standard error (SE) between the replicates (n = 3).

**Figure 3:**
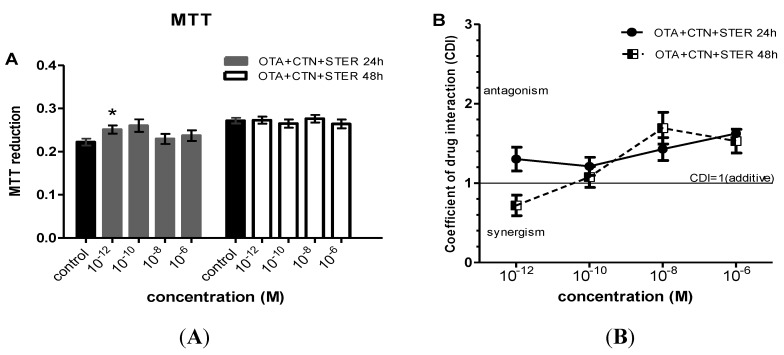
Evaluation of MTT activity in Hep3B cells after co-administration of (**A**) CTN, OTA and STER for 24 and 48h; Bars represent mean values of every concentration at each time point, which was evaluated at three individual experiments, performing measurements at a number of 8-plicates each time (8-plicates, n = 3). Error bars give the standard error (SE) between the replicates (n = 3). The asterisks represent statistically significant differences over the respective vehicle-receiving cells (control) (* *p* < 0.01). CDI values (CDI = 1 CDI < 1 and CDI > 1, shows additive, synergic and antagonistic action respectively) after co-administration of (**B**) CTN, OTA and STER. Again error bars give the standard error (SE) between the replicates (n = 3).

When mycotoxins were administrated in combination, marked increases in MTT metabolic activity were observed at concentrations ranging from 10^−12 ^to 10^−6^ Μ (Figure 2 and Figure 3). In specific, OTA-CTN and OTA-STER co-administration caused statistically significant enhanced MTT reduction at 24 h at concentrations ≥ 10^−10^ M and at 48h at concentrations ≥ 10^−12 ^M (Figure 2C,E). On the other hand, the same effect was less intense after co-exposure to CTN-STER and CTN-STER-OTA, being though statistically significant at 24 h at 10^−12 ^M (Figure 2A and Figure 3A), as OTA’s addition did not modify MTT reducing activity in Hep3B cells in comparison with control cultures.

Using the coefficient of drug interaction (CDI) index, an antagonistic interaction was revealed among all the under research mycotoxins, in particular at concentrations ranging from 10^−8 ^to 10^−6^ Μ, observed at all exposure periods (Figure 2b,d,f and Figure 3b, respectively). Indeed, antagonism at this concentration range reversed the respective STER and CTN toxicity effect presented at single administration schemes, increasing MTT activity in Hep3B cells. At concentrations as low as 10^−12^ Μ all the possible mycotoxin combinations tent to show synergism in their MTT promoting effect at 48 h. The co-exposure of Hep3B cultures to OTA-CTN led to a similar synergistic effect after 24 h as it is being shown on Figure 2D. Interestingly, OTA’s addition did not affect CTN-STER interaction profile nor altered significantly MTT activity in Hep3B cells in comparison with control cultures.

### 3.3. Cytogenetic Effects of CTN, OTA and STER in Hep3B Cells

We evaluated SCE rates, cell cycle kinetics and mitotic divisions after CTN, OTA and STER single or combined administrations. Untreated and vehicle-treated cultures (control groups) exhibit similar SCE rates (0.22 ± 0.02 SCE/chromosome *vs.* 0.20 ± 0.02 SCE/chromosome, respectively, *p* > 0.05), mitotic divisions (MI) (46.2 ± 0.6 ‰ *vs.* 44.6 ± 1.2 ‰, respectively, *p* > 0.05) and cell cycle kinetics (PRI) (2.21 ± 0.03 *vs.* 2.18 ± 0.03, respectively, *p* > 0.05), indicating that the solvent did not affect the above mentioned indices. 

**Figure 4:**
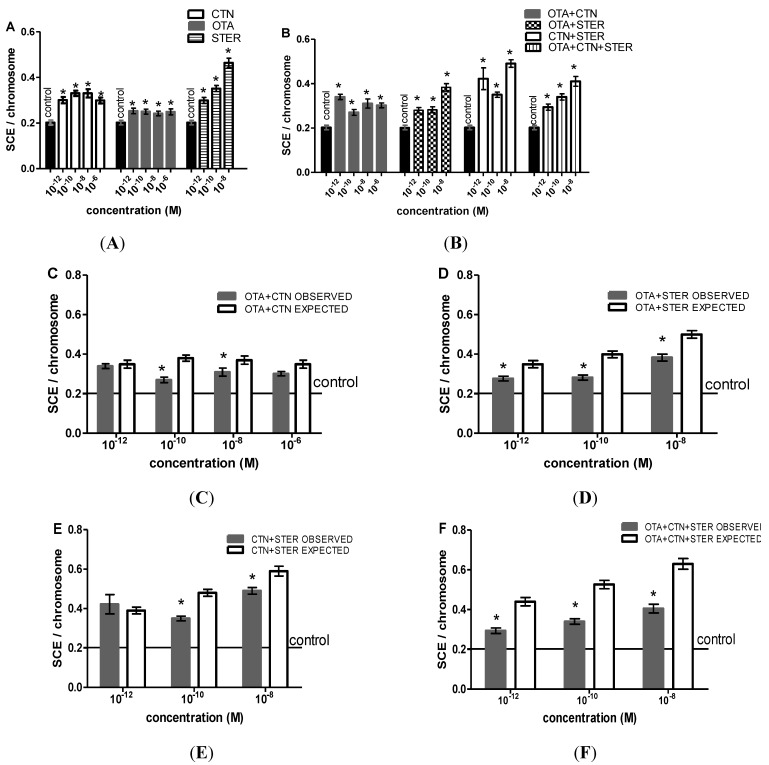
SCE rates after (**A**) single CTN, OTA or STER administration, (**B**) CTN, OTA and STER co-administration. STER at 10^−6 ^M was highly cytotoxic. Bars represent mean ± SEM. All treatments were tested in three independent experiments and approximately 60–75 2nd division metaphases were scored for each treatment. The asterisks indicate statistically significant differences over vehicle-treated cells (*p* < 0.01). Expected *versus* observed SCE rates for (**C**) OTA + CTN, (**D**) OTA + STER, (**E**) CTN + STER and (**F**) OTA + CTN + STER treatments. EV = (OV_1_ + OV_2_ + OV_3_) – (N − 1) × OV_control_, where EV is the expected value of the combined treatment of N substances, OV is the mean observed value of each individual treatment of N substances and OV_control_ is the mean observed value in control cultures. OV = EV, implies an additive, OV > EV, implies a synergistic and OV < EV, implies an antagonistic effect. The asterisks indicate statistically significant differences between OV and ΕV (*p* < 0.05).

STER alone or in combination with OTA and/or CTN caused significant increase in the number of SCE/chromosome compared with the vehicle-treated cells that except for STER + CTN co-treatment exhibited a dose-dependent character (Figure 4A,B). At concentrations as high as 10^−6 ^M STER proved highly cytotoxic so we were unable to determine SCE rates in the respective single or co-treated cultures. Marked increases in SCE rates were also shown after OTA and CTN single and combined administration.

Observed and expected mean values of SCE rates were compared in order to evaluate mycotoxins’ interaction profile as a result of co-administration. A slight though statistically significant antagonistic effect is shown at concentrations ≤ 10^−8 ^M (Figure 4C–F), but still the enhanced SCE frequency observed at single administration schemes is retained. 

**Figure 5:**
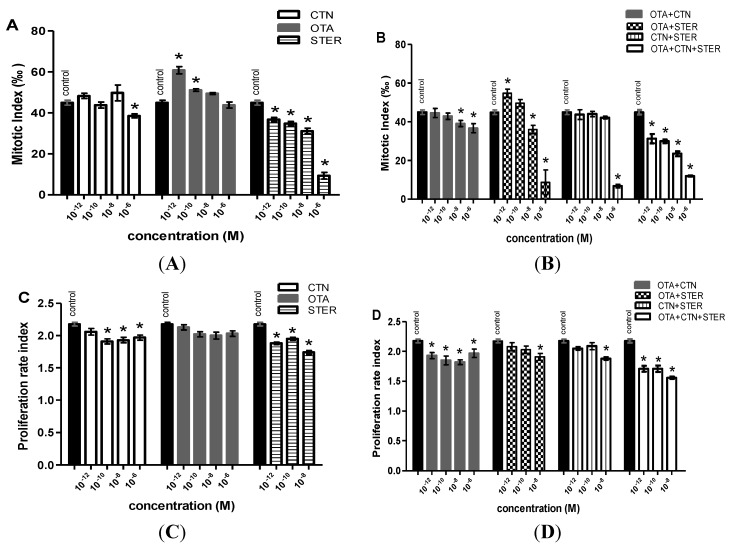
MI differentiation after (**A**) single CTN, OTA or STER administration, (**B**) CTN, OTA and STER co-administration. All treatments were tested in three independent experiments and approximately 12,000 nuclei were scored for each treatment. PRI differentiation after (**C**) single CTN, OTA or STER administration, (**D**) CTN, OTA and STER co-administration. All treatments were tested in three independent experiments and approximately 500–600 metaphases were scored for each treatment. STER and STER-co-treatments at 10^−6 ^M were highly cytotoxic. Bars represent mean ± SEM. The asterisks indicate statistically significant differences over the respective vehicle-treated cells (*p* < 0.01).

Results of MI after single and combined mycotoxin administration are summarized in Figure 5A,B. Apart from OTA, all treatments reduced the MI at concentrations >10^−8 ^M. STER and OTA + CTN + STER were the most potent MI suppressors as they significantly reduced mitotic divisions at all concentrations tested. On the other hand Hep3B exposure to OTA and OTA+STER increases the MI at concentrations ≥ 10^−1^ M (Figure 5A,B). No significant changes were observed between combined and the respective individual treatments. 

Mycotoxins’ effect on the cell-cycle progression is shown in Figure 5C,D. All treatments but OTA caused marked PRI inhibition at concentrations >10^−1^ M. Again STER appeared highly cytotoxic at 10^−6 ^M, so we were unable to determine PRI in the respective single or co-treated cultures. Interestingly, Hep3B exposure to STER or OTA+CTN and OTA+CTN+STER exerted statistically significant PRI reduction at concentrations < 10^−1^ M. No significant changes were observed between individual and combined treatments.

## 4. Discussion

Mycotoxin effects on public health have been extensively studied since chronic exposure can lead to serious health problems, even carcinogenesis [36,37]. It is of great importance to investigate the effects of mycotoxin mixtures rather than focus on individual mycotoxins only, since they frequently coexist. OTA and CTN are the most common concurrent contaminants. Co-occurrence of STER is also expected as fungi strains of *Aspergillus* and *Penicillium*, from which CTN, OTA and STER are mainly produced, usually coexist [29]. The genotoxic and cytotoxic potential of OTA and CTN have been reported *in vivo* and *in vitro*, whereas STER is less studied. The doses in which these toxins are commonly tested are usually from 10^−6 ^to 2 × 10^−4^ M. However their potency at much lower concentrations has also attracted interest, mainly for OTA, as there are indications of cytogenetic and mutagenetic activity after chronic exposure to concentrations as low as 10^−9^ to 10^−12^ M [38,39,40].

Thus, herein, we focus on the *in vitro* genotoxic and cytotoxic effects of STER on the human hepatocellular cancer cell line Hep3B applied at toxic and sub-toxic doses, and in single or combined OTA and/or CTN administration.

The MTT assay is widely used to study mitochondrial activity and consequently cellular metabolism. Changes in metabolic activity usually represent changes in cell viability, as a result of cytotoxic activity. STER and CTN single administration exerted clear toxicity at concentrations >10^−9^ M and reduced the metabolic activity of Hep3B cells at a 1000-fold lower concentration than OTA. Based on IC50 values, these three mycotoxins affect Hep3B cells in the following order STER > OTA > CTN. Similar OTA IC_50_ values (24 h: 90 μΜ, 48 h: 30 μΜ) have been reported in hepatoma cell line HepG2 [41], while STER demonstrated a 50% reduction of neutral red uptake at 286.1 μΜ at 24 h in the same cell line [42]. The differentiation in STER’s IC_50_ value can be attributed to the functional properties of Hep3B and HepG2 cells, as well as to the different solvents and determined parameters (Neutral Red: lysosomal activity, MTT: mitochondrial activity). 

Cytotoxic effects and cell-cycle delays after single mycotoxin administration were also assessed through the MI and PRI analysis, which is indicative of both cytotoxic and cytostatic activity. Hep3B treatment with STER significantly suppressed the two indices. On the other hand, CTN caused minor MI and PRI inhibition, whereas OTA did not significantly affect cell progression neither exerted cytotoxic activity. Overall, these findings demonstrate STER toxicity at pM to μΜ concentrations on Hep3B cells, complying previous studies using different cytotoxicity assays, cell line models and concentrations around 0.5–6 × 10^−6^ M [43]. Concerning CTN, the results obtained from *in vitro* studies for concentrations up to 10^−4 ^Μ support its cytotoxic tendency [44]. For OTA there are *in vitro* reports on bovine lymphocytes, with similar doses with what we herein examine, that also confirm its cytotoxic effect [45]. Slight heterogeneity with our results may be attributed to different metabolic properties and sensitivity of each cell line model.

Effects of the three mycotoxins applied in combination—as naturally occurring—were also addressed. Interestingly, mycotoxin co-administration showed an antagonistic interaction as the inhibitory effect of single dosage was reversed resulting in increased MTT activity. These increases, however, were not confirmed in MI and PRI assays. MTT reduction is often affected from cellular, metabolic and molecular factors that just alter the mitochondrial activity and might significantly impact the evaluation and quantitation of cell viability [46,47]. When applying the MI and PRI assays, all mycotoxin combinations showed clear cytotoxic and cytostatic activity at concentrations >10^−9^ Μ and mainly an additive interaction. It is worth mentioning that STER-OTA-CTN co-administration had the same effect for all the concentrations examined. 

To our knowledge this is the first study dealing with alterations of the MTT, MI and PRI in response to exposure to mycotoxin combinations, *i.e.*, STER with OTA and CTN, providing a better insight on the possible cytotoxic and cytostatic interactions. Previous *in vitro* reports mainly address toxicity effects of CTN-OTA co-exposure using different cytotoxicity tests. For concentrations higher than 10^−6^ M, combinations of OTA and CTN showed clear synergistic cytotoxic effects on green monkey kidney Vero cells and porcine renal cell line LLC-PK1, using the MTT assay [22,39,40]. In V79 hamster fibroblasts and porcine urinary bladder epithelial cells co-treatment with OTA-CTN displayed antagonism as assessed by the Neutral Red uptake assay [48]. The same combination, at doses lower than IC50 for each individual toxin, had additive cytotoxic effects on porcine kidney epithelial cells PK15 when using the Trypan Blue assay and antagonistic, resulting in significantly increased MTT-reducing activity [49]. Therefore, variation involving CTN-OTA interaction and toxic activity is not unexpected as it depends on the *in vitro* model and the cytotoxicity assay when examined at toxic or sub-toxic levels.

In our results, mycotoxins exhibited various interactions depending on the assay performed and the concentration examined. Apart from being metabolites of similar fungi genus and possessing some chemical structure similarities, examining their mechanism of action at a molecular or cellular level is essential for a better understanding of their combined effects. CTN leads to cell death after causing disruption through selective loss of cell membrane permeability. STER as a biogenetic precursor of AFL probably leads to cell deregulation and death through DNA modification. OTA preserves a complex mode of action in comparison with the others toxins. It can affect cell viability by disrupting metabolism and reducing glyconeogenesis or by causing cell deregulation and affecting membrane permeability [50]. Thus, every mycotoxin has a specific biological pathway to affect cell viability and this is probably the reason why they share a complicated interaction profile *in vitro.*

Finally, being aware of the fact that STER, OTA and CTN share properties capable of affecting the cell genome, we employed the SCE assay in order to investigate their genotoxic potency. SCE assay is a rapid and sensitive method to detect DNA damage and evaluate possible environmental mutagens/carcinogens [51]. According to our results, Hep3B cells are sensitized to SCE induction by all mycotoxins even at the lowest concentrations (10^−12^ M). STER demonstrates a potent dose-dependent genotoxic effect while OTA is the least genotoxic. In accordance with our results is an *in vivo* study in mouse bone marrow cells, where STER induced SCE rates and chromosomal aberrations in mouse and rat bone marrow cells, in a dose-dependent manner [52,53]. *In vitro* studies have reported a dose-dependent increase in SCE rates and chromosomal aberrations after OTA treatment [54], while an *in vivo* report has stated the opposite [55]. CTN had no effect on SCE formation in Chinese hamster ovary cells CHO-K1 and human peripheral blood lymphocytes at concentrations ranging from 5 to15 μΜ [56]. Inconsistencies with our results could be attributed to differences in experimental models and protocols.

All combined treatments increase SCE rates, from pM concentrations. Notwithstanding the fact that an antagonistic interaction was prevalent at concentrations ≤ 10^−8 ^M in all the co-administration schemes the already significantly enhanced SCE ratio was maintained. According to previous studies, both OTA and CTN can lead to free radical generation, while free radicals can induce DNA damage and increase SCE rates [57,58,59,60,61,62]. OTA and STER form DNA adducts [63,64]; some types of DNA adducts have also been correlated with SCE formation [65,66]. Based on these findings one would expect that mycotoxin co-treatments would induce SCE rates additively (the least). However, a less-than-additive effect is evident in most combined treatments. It seems that the underlying mechanisms are quite complex and thus, in depth analysis is required. 

## 5. Conclusions

The present study is the first in the field dealing with the cytotoxic and cytogenetic effects of STER, OTA and CTN alone or in mixtures at concentrations relevant or lower than those that have been detected in human serum [24,25,26,27,28]. According to our results, Hep3B single treatment with STER displayed a highly toxic and genotoxic action in comparison with OTA and CTN. All mycotoxin schemes enhanced SCEs even at pM concentrations. STER co-treatment with OTA and/or CTN did not affect the cytogenetic effect presented at single administration despite the increased MTT reducing activity. Mycotoxin co-treatments demonstrate in general similar-to-additive or antagonistic cytotoxic and cytogenetic effects, while SCE induction seems to be regulated by complex mechanisms that need to be clarified. Based on our findings, *in vitro* exposure to STER alone or in combination with OTA and CTN, provides effects that might have an impact on public health, even at pM concentrations.

## References

[1] Peraica M., Radic B., Lucic A., Pavlovic M. (1999). Toxic effects of mycotoxins in humans. Bull. World Health Organ..

[2] Mycotoxins, Risks in Plant, Animal and Human Systems. http://www.trilogylab.com/uploads/Mycotoxin_CAST_Report.pdf.

[3] Chu F.S. (1991). Mycotoxins: Food contamination, mechanism, carcinogenic potential and preventive measures. Mutat. Res..

[4] Bryden W.L. (2007). Mycotoxins in the food chain: Human health implications. Asia Pac. J. Clin. Nutr..

[5] Shephard G.S. (2008). Impact of mycotoxins on human health in developing countries. Food Addit. Contam. Part A Chem. Anal. Control. Expo. Risk Assess..

[6] Wild C.P., Gong Y.Y. (2010). Mycotoxins and human disease: A largely ignored global health issue. Carcinogenesis.

[7] Berthiller F., Schuhmacher R., Adam G., Krska R. (2009). Formation, determination and significance of masked and other conjugated mycotoxins. Anal. Bioanal. Chem..

[8] Versilovskis A., de Saeger S. (2010). Sterigmatocystin: Occurrence in foodstuffs and analytical methods—An overview. Mol. Nutr. Food Res..

[9] Wang J.S., Groopman J.D. (1999). DNA damage by mycotoxins. Mutat. Res..

[10] Scientific Opinion on the Risk for Public and Animal Health Related to the Presence of Sterigmatocystin in Food and Feed. http://www.efsa.europa.eu/en/efsajournal/pub/3254.htm.

[11] Yabe K., Nakajima H. (2004). Enzyme reactions and genes in aflatoxin biosynthesis. Appl. Microbiol. Biotechnol..

[12] International Agency for Research on Cancer (IARC) (1976). Some Naturally Occurring Substances. Monographs on the Evaluation of Carcinogenic Risks to Humans.

[13] International Agency for Research on Cancer (IARC) (1987). Overall Evaluations of Carcinogenicity: An Updating of IARC Monographs Volumes 1 to 42. Monographs on the Evaluation of Carcinogenic Risks to Humans.

[14] Wang J., Huang S., Xing L., Shen H., Yan X., Wang J., Zhang X. (2013). Role of hMLH1 in sterigmatocystin-induced G2 phase arrest in human esophageal epithelial Het-1A cells *in vitro*. Toxicol. Lett..

[15] International Agency for Research on Cancer (IARC) (1993). Some Naturally Occurring Substances: Food Items and Constituents, Heterocyclic Aromatic Amines and Mycotoxins. Monographs on the Evaluation of Carcinogenic Risks to Humans.

[16] Miliccevicc D., Jovanovicc M., Matekalosverak V., Radiccevicc T., Petrovicc M.M., Lilicc S. (2011). A survey of spontaneous occurrence of ochratoxin a residues in chicken tissues and with histopathological changes in liver and kidneys. J. Environ. Sci. Health. C Environ. Carcinog. Ecotoxicol. Rev..

[17] Gupta R.S. (2007). Veterinary Toxicology.

[18] Marquardt R.R., Frohlich A.A. (1992). A Review of recent advances in understanding ochratoxicosis. J. Anim. Sci..

[19] International Agency for Research on Cancer (IARC) (1998). Some Naturally Occurring and Synthetic Food Components, Coumarins and Ultraviolet Radiation. Monographs on the Evaluation of Carcinogenic Risks to Humans.

[20] Scientific Opinion on the Risks for Public Animal and Human Health Related to the Presence of Citrinin in Food and Feed. http://www.efsa.europa.eu/en/efsajournal/doc/2605.pdf.

[21] Vrabcheva T., Usleber E., Dietrich R., Martlbauer E. (2000). Co-occurrence of ochratoxin a and citrinin in cereals from bulgarian villages with a history of balkan endemic nephropathy. J. Agric. Food Chem..

[22] Leszkowicz A.P., Tozlovanu M., Manderville R., Peraica C.M., Stefanovic V. (2007). New molecular and field evidences for the implication of mycotoxins but not aristolochic acid in human nephropathy and urinary tract tumor. Mol. Nutr. Food Res..

[23] Fan L.L., Sun G.P., Wei W., Wang Z.G., Ge L., Fu W.Z., Wang H. (2010). Melatonin and doxorubicin synergistically induse cell apoptosis in human hepatoma cell lines. World J. Gastroenterol..

[24] Hutanasu C., Sfarti C., Trifan A., Cojocariu C., Singeap A.M., Spac A., Stanciu C. (2011). High levels of sterigmatocystinin patients with chronic liver diseases. Rev. Med. Chir. Soc. Med. Nat. Iasi..

[25] Blaszkewicz M., Munoz K., Degen G.H. (2013). Methods for analysis of citrinin in human blood and urine. Arch. Toxicol..

[26] Ozcelik N., Kosar A., Soysal D. (2001). Ochratoxin A in human serum samples collected in Isparta-Turkey from healthy individuals and individuals suffering from different urinary disorders. Toxicol. Lett..

[27] Breitholtz-Emanuelsson A., Minervini F., Hult K., Visconti A. (1994). Ochratoxin A in human serum samples collected in southern Italy from healthy individuals and individuals suffering from different kidney disorders. Nat. Toxins.

[28] Skaug M.A. (2003). Levels of ochratoxin A and IgG against conidia of *Penicillium verrucosum* in blood samples from healthy farm workers. Ann. Agric. Environ. Med..

[29] Prange A., Modrow H., Hormes J., Kramer J., Kohler P. (2005). Influence of Mycotoxin producing fungi (Fusarium, Aspergillus, Penicillium) on gluten proteins during suboptimal storage of wheat after harvest and competitive interactions between field and storage fungi. J. Agric. Food Chem..

[30] Guo L., Dial S., Shi L., Branham W., Liu J., Fang J.L., Green B., Deng H., Kaput J., Ning B. (2011). Similarities and differences in the expression of drug-metabolizing enzymes between human hepatic cell lines and primary human hepatocytes. Drug Metab. Despos..

[31] Mayer B.J., Sundermann V.M., Darroudi F., Laky B., Wit K., Knasmüller S. (2004). Genotoxic effects of dietary and lifestyle related carcinogens in human derived hepatoma (HepG2, Hep3B) cells. Mutat. Res..

[32] Volders M.K., Decordier I., Elhajouji A., Plas G., Aardema M.J., Fenech M. (2011). *In vitro* genotoxicity testing using the micronucleus assay in cell lines, human lymphocytes and 3D human skin models. Review. Mutagenesis.

[33] Perry P., Wolf S. (1974). New Giemsa method for the differential staining of sister chromatids. Nature.

[34] Xu S.P., Sun G.P., Shen Y.X., Peng W.R., Wang H., Wei W. (2007). Synergistic effect of combining paeonol and cisplatin on apoptotic induction of human hepatoma cell lines. Acta. Pharmacol. Sin..

[35] Follmann W., Hillebrand I.E., Creppy E.E., Bolt H.M. (1995). Sister chromatid exchange frequency in cultured isolated porcine urinary bladder epithelial cells (PUBEC) treated with ochratoxin A and alpha. Arch. Toxicol..

[36] Rensburg S.J.V., Mozaffari P.C., Schalkwyk D.J.V., Watt J.J.V.D., Vincent T.J., Purchase I.F. (1985). Hepatocellular carcinoma and dietary aflatoxin in Mozambique and Transkei. Br. J. Cancer.

[37] Wogan G.S., Hecht S.S., Felton J.S., Conney A.H., Loeb L.A. (2004). Environmental and chemical carcinogenesis. Semin. Cancer Biol..

[38] Weber F., Freudinger R., Schwerdt G., Gekle M. (2005). A rapid screening method to test apoptotic synergisms of ochratoxin A with other nephrotoxic substances. Toxicol. In Vitro.

[39] Bouslimi A., Bouaziz C., Boussema I.A., Hassen W., Bacha H. (2008). Individual and combined effects of ochratoxin A and citrinin on viability and DNA fragmentation in cultured Vero cells and on chromosome aberrations in mice bone marrow cells. Toxicology.

[40] Heussner A.H., Dietrich D.R., Brien E.O. (2006). In vitro investigation of individual and combined cytotoxic effects of ochratoxin A and other selected mycotoxins on renal cells. Toxicol. In Vitro.

[41] Bouaziz C., Dein O.S., Golli E., Essefi S.A., Brenner C., Lemaire C., Bach H. (2008). Different apoptotic pathways induced by zearalenone, T-2 toxin and ochratoxin A in human hepatoma cells. Toxicology.

[42] Bunger J., Westphal G., Monnich A., Hinnendahl B., Hallier E., Muller M. (2004). Cytotoxicity of occupationally and environmentally relevant mycotoxins. Toxicology.

[43] Jaksic D., Puel O., Canlet C., Kopjar N., Kosalec I., Klaric M.S. (2012). Cytotoxicity and genotoxicity of versicolorins and 5-methoxysterigmatocystin in A549 cells. Arch. Toxicol..

[44] Chang C.H., Yu F.Y., Wu T.S., Wang L.T., Liu B.H. (2011). Mycotoxin Citrinin induced cell cycle G2/m arrest and numerical chromosomal aberration associated with disruption of microtubule formation in human cells. Toxicol. Sci..

[45] Lioi M.B., Santoro A., Barbieri R., Salzano S., Ursini M.V. (2004). Ochratoxin A and zearalenone: A comparative study on genotoxic effects and cell death induced in bovine lymphocytes. Mutat. Res..

[46] Bernhard D., Schwaiger W., Crazzolara R., Tinhofer I., Kofler R., Csordas A. (2003). Enhanced MTT-reducing activity under growth inhibition by resveratrol in CEM-C7H2 lymphocytic leukemia cells. Cancer Lett..

[47] Vistica D.T., Skehan P., Scudiero D., Monks A., Pittman A., Boyd M.R. (1991). Tetrazolium-based assays for cellular viability: A critical examination of selected parameters affecting formazan production. Cancer Res..

[48] Follmann W., Lebrun S., Kullik B., Koch M., Romer H.C., Golka K. (2000). Cytotoxicity of ochratoxin A and citrinin in different cell types in vitro. Mycotoxin. Res..

[49] Klaric M.S., Zeljezic D., Rumora L., Peraica M., Pepeljnjak S., Domijan A.M. (2012). A potential role of calcium in apoptosis and aberrant chromatin forms in porcine kidney PK15 cells induced by individual and combined ochratoxin A and citrinin. Arch. Toxicol..

[50] Speijers G.J.A., Speijers M.H.M. (2004). Combined toxic effects of mycotoxins. Toxicol. Lett..

[51] Bradley M.O., Hsu I.C., Harris C.C. (1979). Relationship between sister chromatid exchange and mutagenicity, toxicity and DNA damage. Nature.

[52] Curry P.T., Reed R.N., Martino R.M., Kitchin R.M. (1984). Induction of sister-chromatid exchanges in vivo in mice by the mycotoxins sterigmatocystin and griseofulvin. Mutat. Res..

[53] Ueda N., Fujie K., Mimura K.G., Chattopadhyay S.C., Sugiyama T. (1984). Acute cytogenetic effect of sterigmatocystin on rat bone-marrow cells in vivo. Mutat. Res..

[54] Hennig A., Fink-Gremmels J., Leistner L. (1991). Mutagenicity and effects of ochratoxin A on the frequency of sister chromatid exchange after metabolic activation. IARC Sci Publ..

[55] Bendele A.M., Neal S.B., Oberly T.J., Thompson C.Z., Bewsey B.J., Hill L.E., Rexroat M.A., Carlton W.W., Probst G.S. (1985). Evaluation of ochratoxin A for mutagenicity in a battery of bacterial and mammalian cell assays. Food Chem. Toxicol..

[56] Liu B.H., Yu F.Y., Wu T.S., Li S.Y., Su M.C., Wang M.C., Shiha S.M. (2003). Evaluation of genotoxic risk and oxidative DNA damage in mammalian cells exposed to mycotoxins, patulin and citrinin. Toxicol. Appl. Pharmacol..

[57] Cavin C., Delatour T., Marin-Kuan M., Fenaille F., Holzhäuser D., Guignard G., Bezencon C., Piguet D., Parisod V., Richoz-Payot J. (2009). Ochratoxin A-mediated DNA and protein damage: Roles of nitrosative and oxidative stresses. Toxicol. Sci..

[58] Liu J., Wang Y., Cui J., Xing L., Shen H., Wu S., Lian H., Wang J., Yan X., Zhang X. (2012). Ochratoxin A induces oxidative DNA damage and G1 phase arrest in human peripheral blood mononuclear cells in vitro. Toxicol. Lett..

[59] Chen C.C., Chan W.H. (2009). Inhibition of citrinin-induced apoptotic biochemical signaling in human hepatoma G2 cells by resveratrol. Int. J. Mol. Sci..

[60] Cooke M.S., Evans M.D., Dizdaroglu M., Lunec J. (2003). Oxidative DNA damage: Mechanisms, mutation, and disease. FASEB J..

[61] Nicotera T.M., Notaro J., Notaro S., Schumer J., Sandberg A.A. (1989). Elevated superoxide dismutase in bloom’s syndrome: A genetic condition of oxidative stress. Cancer Res..

[62] Schaaf G.J., Nijmeijer S.M., Maas R.F., Roestenberg P., de Groene E.M., Fink-Gremmels J. (1588). The role of oxidative stress in the ochratoxin A-mediated toxicity in proximal tubular cells. Biochim. Biophys. Acta.

[63] McConnell I.R., Garner R.C. (1994). DNA adducts of aflatoxins, sterigmatocystin and other mycotoxins. IARC Sci. Publ..

[64] Pfohl-Leszkowicz A., Grosse Y., Kane A., Creppy E.E., Dirheimer G. (1993). Differential DNA adduct formation and disappearance in three mouse tissues after treatment with the mycotoxin ochratoxin A. Mutat. Res..

[65] Heflich R.H., Morris S.M., Beranek D.T., McGarrity L.J., Chen J.J., Beland F.A. (1986). Relationships between the DNA adducts and the mutations and sister chromatid exchanges produced in Chinese hamster ovary cells by N-hydroxy-2-aminofluorene, N-hydroxy-N’-acetylbenzidine and 1-nitrosopyrene. Mutagenesis.

[66] Heflich R.H., Beranek D.T., Kodell R.L., Morris S.M. (1982). Induction of mutations and sister-chromatid exchanges in Chinese hamster ovary cells by ethylating agents. Mutat. Res..

